# Association between natriuretic peptides and C-reactive protein with frailty in heart failure: a systematic review and meta-analysis

**DOI:** 10.1007/s40520-024-02713-x

**Published:** 2024-03-06

**Authors:** Konstantinos Prokopidis, Hironori Ishiguchi, Cara Jordan, Krzysztof Irlik, Katarzyna Nabrdalik, Francesc Formiga, Rajiv Sankaranarayanan, Gregory Y. H. Lip, Masoud Isanejad

**Affiliations:** 1 Department of Musculoskeletal Ageing and Science, Institute of Life Course and Medical Sciences, University of Liverpool, Liverpool, UK; 2 Liverpool Centre for Cardiovascular Science at University of Liverpool, Liverpool John Moores University and Liverpool Heart and Chest Hospital, Liverpool, UK; 3 Division of Cardiology, Department of Medicine and Clinical Science, Yamaguchi University Graduate School of Medicine, Ube, Japan; 4 School of Health and Life Sciences, Teesside University, Middlesbrough, UK; 5 Faculty of Medical Sciences in Zabrze, Students’ Scientific Association By the Department of Internal Medicine, Diabetology and Nephrology in Zabrze, Medical University of Silesia, Katowice, Poland; 6 Doctoral School, Faculty of Medical Sciences in Zabrze, Department of Internal Medicine, Diabetology and Nephrology, Medical University of Silesia, Katowice, Poland; 7 Faculty of Medical Sciences in Zabrze, Department of Internal Medicine, Diabetology and Nephrology, Medical University of Silesia, Katowice, Poland; 8 Servicio de Medicina Interna, Hospital Universitari de Bellvitge, Barcelona, Spain; 9 Liverpool University Hospitals NHS Foundation Trust, Liverpool, UK; 10 National Institute for Health and Care Research, Liverpool, UK; 11 Danish Center for Clinical Health Services Research, Department of Clinical Medicine, Aalborg University, Aalborg, Denmark

**Keywords:** Heart failure, Frailty, BNP, NT-proBNP, CRP

## Abstract

**Background:**

Heart failure (HF) and frailty are accompanied by a bidirectional relationship, sharing common risk factors including elevated levels of natriuretic peptides and inflammation. The aim of this study was to compare biomarkers associated with poor clinical outcomes, that is, plasma brain natriuretic peptide (BNP), N-terminal-pro B-type natriuretic peptide (NT-proBNP), and C-reactive protein (CRP) in patients with HF and frailty vs. patients with HF without frailty.

**Methods:**

From inception until July 2023, PubMed, Scopus, Web of Science, and Cochrane Library a systematic literature search was conducted. To evaluate whether frailty is linked with greater levels of BNP, NT-proBNP, and CRP, a meta-analysis using a random-effects model was used to calculate the pooled effects (CRD42023446607).

**Results:**

Fifty-three studies were included in this systematic review and meta-analysis. Patients with HF and frailty displayed significantly higher levels of BNP (*k* = 11; SMD: 0.53, 95%CI 0.30–0.76, I^2^ = 86%, *P* < 0.01), NT-proBNP (*k* = 23; SMD: 0.33, 95%CI 0.25–0.40, I^2^ = 72%, *P* < 0.01), and CRP (*k* = 8; SMD: 0.30, 95%CI 0.12–0.48, I^2^ = 62%, *P* < 0.01) vs. patients with HF without frailty. Using meta-regression, body mass index (BMI) and age were deemed potential moderators of these findings.

**Conclusions:**

Frailty in HF is linked to increased concentrations of BNP, NT-proBNP, and CRP, which have been epidemiologically associated with adverse outcomes. The increased risk of NYHA III/IV classification further emphasizes the clinical impact of frailty in this population.

**Supplementary Information:**

The online version contains supplementary material available at 10.1007/s40520-024-02713-x.

## Introduction

Frailty is a dynamic, multidimensional syndrome with an increased risk of presentation with advancing age. It is characterized by an increased vulnerability to external stressors and thus an increased risk of adverse health outcomes [1]. There are currently various different approaches of frailty diagnosis used in clinical practice, on one hand those that define frailty as a risk physical phenotype preceding dependency and on the other hand those that value frailty as an accumulation of deficits (multidimensional frailty), including comorbidities, disabilities, symptoms, and biochemical markers [2].

Heart failure (HF) is a clinical disorder marked by structural and/or functional myocardial abnormalities that result in high intracardiac pressure and insufficient cardiac output [3]. Interestingly, patients with HF display higher inflammatory status [4] and major skeletal muscle abnormalities, including a shift in muscle fibre type distribution with fewer type II muscle fibres and a lower capillary-to-fiber ratio, which may contribute to exercise intolerance and accelerated losses of muscle mass and function [5]. The presence of HF may accelerate the development of frailty with an estimated prevalence of approximately 45% [6].

A biomarker that is associated with HF severity by reflecting mechanical overload and cardiac function is plasma brain natriuretic peptide (BNP), for which research has shown that may be exacerbated by frailty [7]. Likewise, N-terminal-pro B-type natriuretic peptide (NT-proBNP) is a biologically inactive derivative of BNP; a marker commonly used to assess HF severity [8]. In addition, clinical risk stratification for HF has been performed via assessment of the New York Heart Association (NYHA) classification, although it has been deemed an unreliable predictor of negative outcomes in HF, poorly distinguishing patients across a range of functional disabilities [9]. The association between inflammation and HF has been consistent over time, in both clinical and basic research [10].

Considering the negative impact of frailty on HF outcomes and vice versa, it is important to know this potential relationship and examine the degree by which natriuretic peptides, C-reactive protein (CRP), and NYHA scores may differ in patients with HF with or without frailty. In this systematic review and meta-analysis, we aim to compare the differences in plasma BNP, NT-proBNP, CRP, and NYHA classification, in patients with HF and frailty vs. patients with HF without frailty.

## Methods

The revised 2020 Preferred Reporting Items for Systematic Reviews and Meta-Analyses (PRISMA) guidelines were followed to conduct this systematic review and meta-analysis [11]. The protocol has been registered in the International Prospective Register of Systematic Reviews (PROSPERO) (CRD42023446607).

### Search strategy

From the beginning until July 2023, PubMed, Scopus, Web of Science, and Cochrane Library were searched independently by KP and KI. The search phrases “(heart failure OR ejection fraction) AND frail*” were employed. All article duplicates were removed prior to screening.

### Inclusion and exclusion criteria

Studies were included based on the following criteria: (i) data from observational studies (i.e., cross-sectional, longitudinal, and case–control); (ii) patients with HF irrespective of ejection fraction and clinical setting (i.e., inpatients or outpatients); (iii) patients aged ≥ 18 years; (iv) studies including data related to BNP, NT-proBNP, CRP, and NYHA classification for both patients with and without frailty; and (iv) the following criteria for the definition of frailty: Fried’s criteria, Clinical Frailty Scale, FRAIL scale, Rockwood index. Published articles were excluded if they (i) included participants with terminal conditions (i.e., end-stage cancer); (ii) criteria for frailty not specified in inclusion criteria; (iii) were reviews, letters, in vivo or in vitro experiments, commentaries, or posters; and (iv) were not published as a full text and in English.

### Data extraction and risk of bias

Two authors (KP and KI) extracted data independently, which included the name of the first author, year of publication, country of origin, study design, definition of frailty, patient characteristics (sample size, age, gender, body mass index (BMI)), left ventricular ejection fraction (LVEF) rate, and reported comorbidities. Disagreements between authors were resolved by a third investigator (KN).

The quality of the included studies was evaluated using the Methodological index for non-randomized studies (MINORS) tool [12] and performed by two independent reviewers (KP and CJ). MINORS is a comprehensive tool used to assess bias in nonrandomized controlled trials based on the following items: a clearly stated aim; inclusion of consecutive patients; prospective data collection; endpoints appropriate to study aim; unbiased assessment of study endpoint; follow-up period appropriate to study aim; < 5% lost to follow-up; prospective calculation of study size; adequate control group; contemporary groups; baseline equivalence of groups; and adequate statistical analyses. According to the scoring system, MINORS’ domains are scored as 0 if they are not reported, 1 when they have been reported but with inadequate details, and 2 when they have been reported while providing adequate information. The global ideal score is 16 for noncomparative studies, and scores below 8 and 10 were deemed as a high risk of bias and of some concerns, respectively.

### Statistical analysis

Quantitative data were treated as continuous measurements, and changes in outcomes from sarcopenic and non-sarcopenic individuals were compared between groups to calculate standardised mean differences (SMDs) for the evaluation of differences between groups in relation to BNP, NT-proBNP, and CRP, and the odds ratio (OR) regarding the prevalence of NYHA III and IV levels. SMDs were used due to potentially different methods of assessment, which were not described in the respective manuscripts. When studies provided interquartile ranges (IQR), the formula ‘standard deviation (SD) = width of IQR/1.35’ was used to approximately calculate the missing SDs [13]. Statistical significance was assessed using the random-effects model and inverse-variance method.

Statistical heterogeneity of outcome measurements between different studies was assessed using the overlap of their confidence interval (95% CI) and expressed as measurements of Cochran’s Q (Chi-square test) and I^2^. The classification of data as having low heterogeneity was based on I^2^ from 30 to 49%, moderate heterogeneity from 50 to 74% and high heterogeneity from 75% and above [14]. In case of high heterogeneity, meta-regressions were performed using a random-effects model [15] based on BMI, LVEF rate, and age, using STATA/MP 13.0. Subgroup analysis according to different definitions of frailty was also performed. Sensitivity analyses were conducted based on differences in health status (different reported comorbidities between patients with frailty vs. patients without frailty), and studies with increased bias risk. The meta-analysis was synthesized using Review Manager (RevMan 5.4.1) software. A P value of < 0.05 was considered statistically significant.

## Results

### Search results

**Fig. 1:**
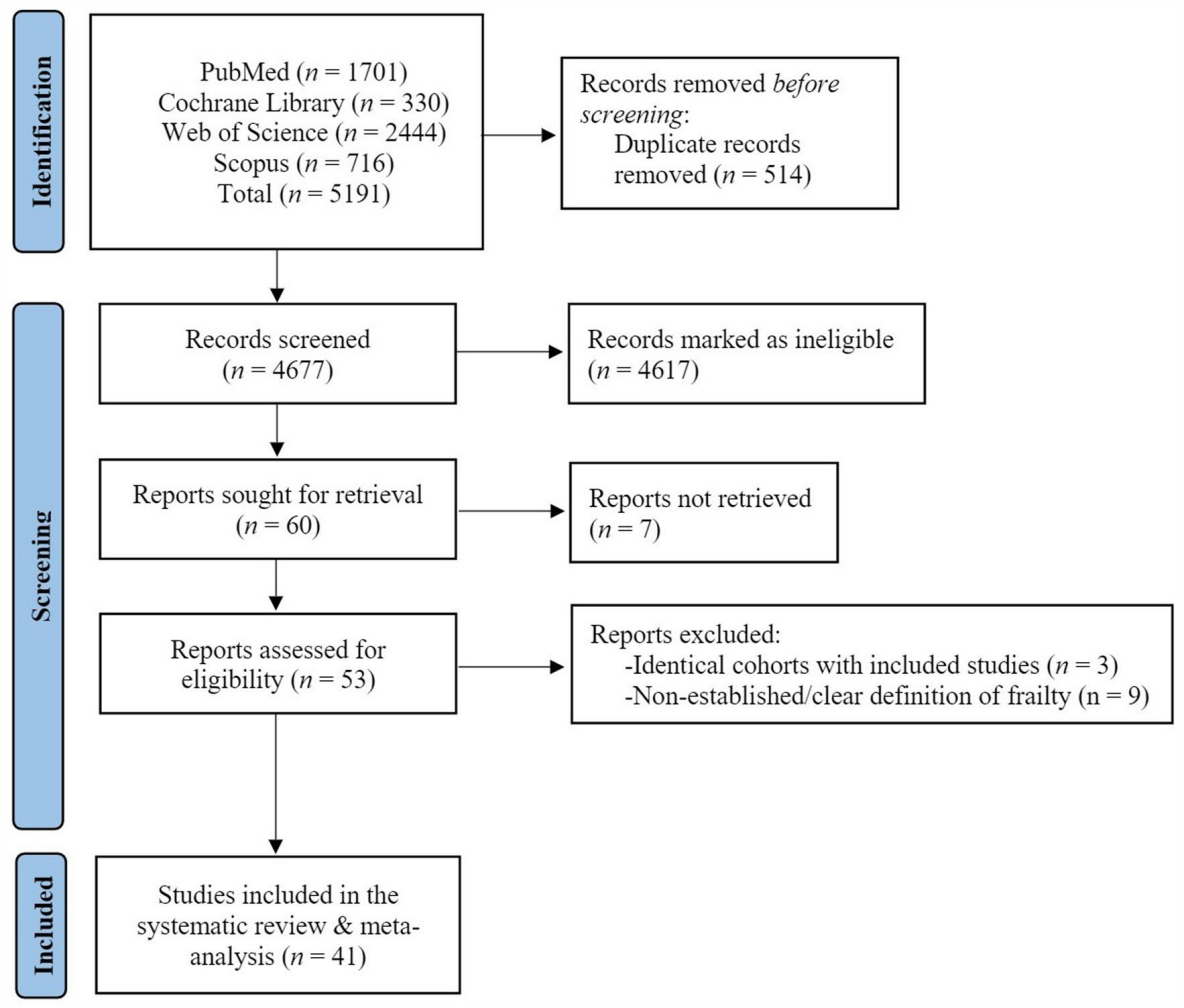
Study flowchart

A flow diagram of the selection process is shown in Fig. 1. The initial literature search provided 5191 publications. Following the exclusion of duplicates and abstracts, 53 full texts were identified as eligible for inclusion in the systematic review and meta-analysis. Of these 53 studies, three studies were excluded due to the inclusion of identical, but more recent or more appropriate cohorts that had already been included in our study [16–18] and nine studies because of the usage of non-established or non-clear frailty definition [19–27]. Overall, 41 studies [7, 18, 24, 28–65] were included in the systematic review and meta-analysis (Fig. 1). Characteristics of the included studies are summarised in Table 1.

**Table 1:** Study and participant characteristics of the included studies assessing frailty

Study, year	Country	Frailty Definition	Total *n*	Frail				Non-frail				Reported comorbidity status
				n (M/F)	Age	BMI	LVEF%	n (M/F)	Age	BMI	LVEF%	
Abe et al*.* 2023	Japan	Fried	1021	604 (362/242)	79 (73–85)	20.8 (19.0–22.6)	33 (26–41)	417 (288/129)	76 (71–81)	21.7 (20.1–23.3)	33 (27–40)	Similar
Aguilar-Iglesias et al*.* 2023	Spain	FRAIL scale	109	68 (40/28)	80 ± 8.7	–	47.4 ± 16.7	41 (25/16)	67 ± 12.8	–	45.2 ± 16.2	Frail had more AF, HT, COPD, CKD
Aguilar-Iglesias et al*.* 2022 (old)	Spain	FRAIL scale	99	51 (29/22)	84 ± 5	–	–	48 (23/25)	82 ± 5	–	–	Frail had more HT, T2D
Aguilar-Iglesias et al*.* 2022 (young)	Spain	FRAIL scale	102	17 (11/6)	68 ± 5	–	–	85 (60/25)	62 ± 9	–	–	Frail had more HT, T2D
Archer et al*. 2*023	USA	Fried	115	49 (16/33)	66.3 ± 15.1	33.8 ± 9.1	46.9 ± 15.9	66 (43/23)	61.5 ± 15.9	29.4 ± 7.1	39.9 ± 15.2	Frail had more CKD, T2D
Ashikawa et al*.* 2022	Japan	Fried	489	130 (80/50)	80 (74–85)	22.8 (20.1–24.7)	–	359 (274/85)	75 (72–79)	22.6 (21.0–24.8)	–	Similar
Boxer et al*.* 2008	USA	Fried	48	15	83 ± 10	28.8 ± 6.1	–	33	73 ± 8	27.7 ± 4.9	–	Similar
Butt et al*.* 2022	Multicenter	Rockwood	3845	1491 (841/650)	72.7 ± 8.8	32.1 ± 6.2	54.1 ± 8.3	2354 (1308/1046)	70.1 ± 10.3	28.1 ± 5.8	54.2 ± 9.1	Frail had more AF, Stroke, MI, Angina, CAD, PAD, VHD, HT, T2D, COPD, Gout, Neuropathy, DLP, Osteoporosis
Chi et al*.* 2022	China	TFI	342	187 (127/60)	–	–	46.36 ± 13.98	155 (107/48)	–	–	48.69 ± 12.94	Frail had a higher number of comorbidities
Denfeld et al*.* 2017	USA	Fried	49	24 (14/10)	60.1 ± 6.4	29.5 ± 6.9	23.3 ± 10.7	25 (19/6)	54.8 ± 11.7	31.0 ± 8.4	25.2 ± 6.8	Similar
Dewan et al*.* 2020	Multicenter	Rockwood	8495	3613 (2731/882)	67.1 ± 10.3	29.1 (25.7–33.0)	29.8 ± 5.8	4882 (3989/893)	61.0 ± 11.7	26.4 (23.7–29.6)	28.5 ± 6.1	Frail had more HT, T2D, AF, VHD, Angina, MI, Stroke, PAD, COPD, Renal disease
Jimenez-Mendez et al*.* 2022	Spain	FRAIL scale	255	111 (47/64)	82.9 ± 4.51	28.6 ± 5.10	46.5 ± 14.5	144 (111/33)	80.2 ± 3.69	27.3 ± 4.38	40.7 ± 13.6	Frail had more HT, CKD
Kałuzna-Oleksy et al*.* 2021	Poland	SHARE-FI	153	52 (41/11)	59.1 ± 8.6	28.0 ± 5.5	22.3 ± 8.1	101 (84/17)	53.1 ± 12.4	29.1 ± 5.7	24.9 ± 7.8	Frail had more CKD
Kanenawa et al*.* 2021	Japan	Clinical Frailty Scale	366	232 (161/71)	83.4 ± 9.1	22.1 ± 3.8	49.7 ± 14.4	134 (65/69)	69.5 ± 11.6	24.1 ± 4.1	42.6 ± 15.4	Non-frail had more Liver Cirrhosis; Frail had more Dementia, CKD
Kaul et al. 2023	Multicenter	Fried	461	259 (112/147)	73.4 ± 8.8	31.6 ± 6.4	57.1 ± 8.3	202 (121/81)	72.4 ± 10.3	30 ± 5.8	55.9 ± 8.0	Frail had more T2D
Khan et al. 2022	Muliticenter	Rockwood	658	495 (326/169)	64 (54–72)	29.4 (24.8–34.9)	25 (5–40)	163 (119/44)	58 (48–68)	27.6 (24.3–31.6)	20 (4–40)	Frail had more COPD, T2D, AF, PVD, Kidney disease, Stroke
Kleipool et al. 2020	The Netherlands	Fried	78	42 (19/23)	81 ± 7.8	–	–	36 (25/11)	71 ± 7.4	–	–	Frail had more T2D
Komici et al. 2020	Italy	Clinical Frailty Scale	128	54 (45/9)	70.5 ± 5.4	24.5 ± 4.6	26.7 ± 6.1	74 (66/8)	68.2 ± 4.2	26.0 ± 4.2	30.2 ± 10.2	Similar
Kondo et al. 2023	Japan	Fried	542	171 (107/64)	81 (74–86)	20.5 (18.4–22.9)	31.0 (25.2–35.0)	371 (308/63)	61 (51–69)	23.5 (21.1–26.9)	28.0 (23.0–33.0)	Frail had more T2D, COPD
Kusunose et al. 2018	Japan	Fried	74	38 (25/13)	79 ± 6	23 ± 3	62 ± 8	36 (19/17)	72 ± 6	24 ± 3	64 ± 7	Similar
Lala et al. 2022	USA	Fried	206	57 (28/29)	61 (56–67)	32.7 (27.5–37.1)	22 (18 –30)	149 (120/29)	62 (53–68)	28.4 (24.9–32.5)	20 (15–25)	Similar
Martin-Sanchez et al. 2017	Spain	Fried	465	169 (44/125)	84.8 ± 6.5	–	–	296 (138/158)	81.1 ± 7.2	–	–	Frail had more HT
Matsuda et al. 2021	Japan	Clinical Frailty Scale	106	90 (47/43)	81 (76–85)	22 ± 4	59 ± 14	16 (12/4)	51 (38–65)	23 ± 4	63 ± 10	Frail had more T2D, Hemodialysis, HT, Stroke, CAD
McDonagh et al. 2023	Australia	Fried	131	71 (48/23)	54 ± 13	27 ± 5	30 ± 16	60 (51/9)	53 ± 16	28 ± 6	31 ± 16	-
Meng et al. 2023	China	Fried	520	145 (62/83)	78.5 ± 6.3	24.6 ± 3.6	63.3 ± 4.4	375 (160/215)	74.3 ± 6.2	25.7 ± 3.3	63.4 ± 4.3	Frail had more Osteoporosis, Stroke, CKD
Metze et al. 2017	Germany	Fried	213	97 (49/48)	79 ± 7	26.2 ± 5.0	–	116 (73/43)	76 ± 9	25.5 ± 4.6	–	Similar
Moayedi et al. 2017	Canada	Fried	100	41 (30/11)	55.8 ± 10.5	26.5 ± 5.7	25.6 ± 11.7	59 (44/15)	51.9 ± 12.1	27.3 ± 5.5	33.6 ± 13.6	Frail had more T2D
Mollar et al*.* 2022	Spain	Fried	182	121 (58/63)	76 ± 10	-	49 ± 15	61 (42/19)	70 ± 12	-	44 ± 15	Similar
Nishiguchi et al*.* 2016	Japan	Fried	206	34 (23/11)	79.2 ± 7.8	22.7 ± 3.2	–	172 (120/52)	72.6 ± 6.7	23.7 ± 3.3	–	Frail had more VHD
Nozaki et al*.* 2020	Japan	Fried	387	207 (118/89)	76.6 ± 6.1	20.9 ± 3.3	47.4 ± 15.9	180 (128/52)	73.1 ± 5.6	22.3 ± 3.0	45.8 ± 15.7	Frail had more T2D
Nozaki et al*.* 2021	Japan	FRAIL scale	537	459 (263/196)	82 (75–86)	20.7 (18.7–23.4)	45 (31–60)	78 (57/21)	76 (68–82)	21.9 (19.1–24.6)	42 (30–55)	Frail had more T2D
Rech et al*.* 2022	Brazil	Fried	15	6 (0/6)	67.7 ± 8.2	30.1 ± 8.0	–	9 (7/2)	66.1 ± 3.9	28.2 ± 3.1	–	–
Ribeiro et al*.* 2021	Brazil	Fried	76	64 (45/19)	70.0 (63.0–75.0)	26.2 ± 4.6	34.5 (10.7)	12 (7/5)	66.0 (62.3–71.3)	28.3 ± 3.9	32.8 ± 11.5	Similar
Rodriguez-Pascual et al*.* 2017	Spain	Fried	497	286 (93/193)	85.7 ± 5.1	–	–	211 (101/110)	84.4 ± 9.4	–	–	Similar
Sanders et al*.* 2018	Multicenter	Rockwood	37.8 (33.6–43.7)	227 (119/108)	69 ± 9	37.8 (33.6–43.7)	58 (55–62)	482 (268/214)	73 ± 10	29.4 (26.1–34.4)	58 (50–65)	Frail had more MI, HT, T2D
Sunaga et al*.* 2021	Multicenter	Clinical Frailty Scale	842	406 (142/264)	85 (81–89)	22.9 (20.4–26.8)	60–65	436 (235/201)	79 (74–84)	24.2 (21.6–26.9)	60–65	No Frail had more DLP
Sze et al*.* 2021	UK	Clinical Frailty Scale	467	206 (124/82)	80 (74–85)	28.4 (24.2–32.4)	–	261 (188/73)	72 (65–79)	29.3 (26.0–34.2)	–	Frail had more PVD, AF, Dementia, COPD, Depression, Anemia
Testa et al*.* 2020	Italy	Fried	112	81 (40/41)	81.1 ± 6.2	26.1 ± 4.4	–	31 (20/11)	78.8 ± 7.3	30.3 ± 10.7	–	Similar
Uzun et al*.* 2022	Turkey	Fried	48	26 (21/5)	56 ± 10	24 ± 4	18 ± 6	22 (19/3)	53 ± 11	27 ± 3	20 ± 5	Similar
Vidan et al*.* 2016	Spain	Fried	416	316 (139/177)	80.8 ± 6.0	27.1 ± 5.6	–	100 (71/29)	77.87 ± 5.6	26.9 ± 5.1	–	Similar
Villarreal et al*.* 2023	Colombia	FRAIL scale	112	68 (41/27)	75.8 ± 11.3	–	44.2 ± 14.5	44 (31/13)	70.6 ± 11.5	–	46.2 ± 15.0	Frail had more HT, CKD
Wang et al*.* 2023	China	Fried	75	29 (27/2)	86.5 ± 5.4	22.7 ± 3.5	56.9 ± 5.2	46 (43/3)	84.2 ± 6.0	24.0 ± 3.0	58.1 ± 5.2	–
Woo et al*.* 2019	China	FRAIL scale	199	95 (15/80)	79.1 ± 7.7	–	–	104 (60/44)	71.8 ± 5.7	–	–	Frail had more T2D, HT

*AF* Atrial fibrillation, *BMI* Body mass index, *CKD* Chronic kidney disease, *CAD* Coronary artery disease, *COPD* Chronic obstructive pulmonary disease, *DLP* Dyslipidemia, *F* Females, *HT* Hypertension, *LVEF* Left ventricular ejection fraction, *M* Male, *MI* Myocardial infarction, *PAD* Peripheral artery disease, *PVD* Peripheral vascular disease, *T2D* Type 2 diabetes, *VHD* Valvular heart disease

Data are expressed as mean (standard deviation) or median (IQR)

### BNP levels in patients with heart failure and frailty versus without frailty

**Fig. 2:**
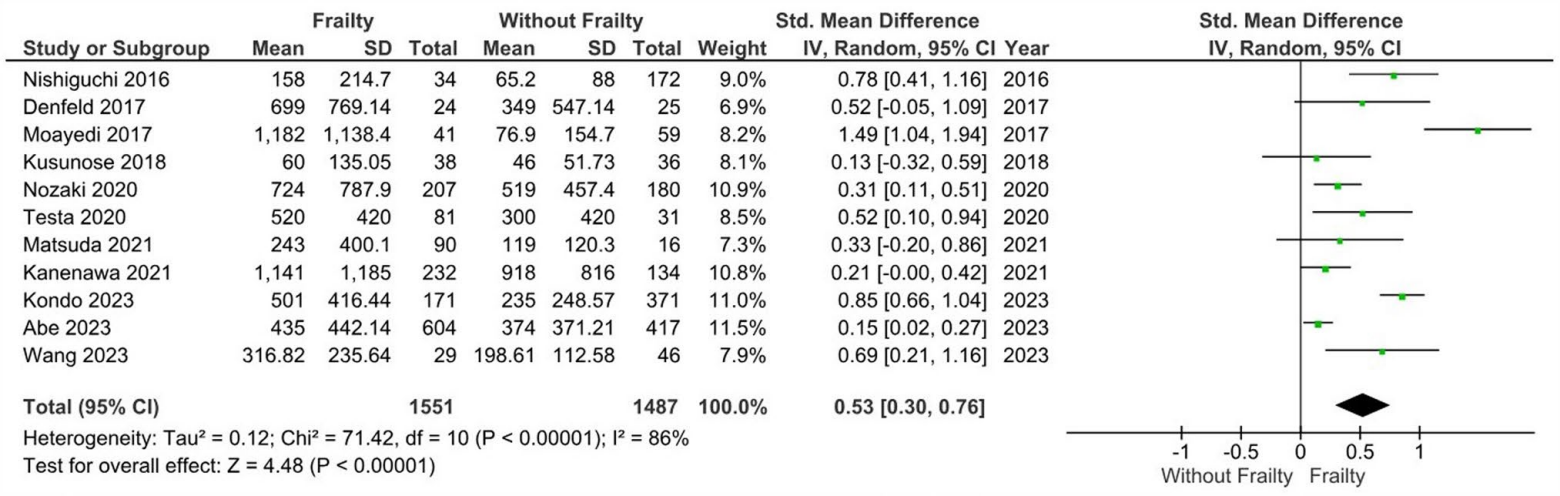
Mean differences in BNP levels according to frailty status in HF patients. Mean differences are presented with 95% confidence intervals using random effects model

Patients with heart failure HF and frailty (*n* = 1551) had significantly higher levels of BNP vs. those without frailty (*n* = 1487), albeit a high degree of heterogeneity was observed (*k* = 11; SMD: 0.53, 95%CI 0.30–0.76, I^2^ = 86%, *P* < 0.01) (Fig. 2). Subgroup analysis based on Fried (*k* = 9; SMD: 0.59, 95%CI 0.31–0.87, I^2^ = 88%, *P* < 0.01) and Clinical Frailty Scale (CFS) criteria (*k* = 2; SMD: 0.23, 95%CI 0.03–0.42, I^2^ = 0%, *P* = 0.03) (Figure S1) showed identical statistical outcomes. Our sensitivity analysis excluding studies in which patients with frailty had increased reported comorbidities revealed similar results (*k* = 5; SMD: 0.34, 95%CI 0.10–0.58, I^2^ = 51%, *P* < 0.01) (Figure S2), while when we evaluated similar health status alongside similar frailty definition criteria (Fried criteria in this case), we also found statistically significant differences (*k* = 5; SMD: 0.34, 95%CI 0.10–0.58, I^2^ = 51%, *P* < 0.01) (Figure S3). Sensitivity analysis based on studies with a high risk of bias did not alter the findings from the main analysis (*k* = 8; SMD: 0.53, 95%CI 0.23–0.84, I^2^ = 89%, *P* < 0.01) (Figure S4).

### NT-proBNP levels in patients with heart failure and frailty versus without frailty

**Fig. 3:**
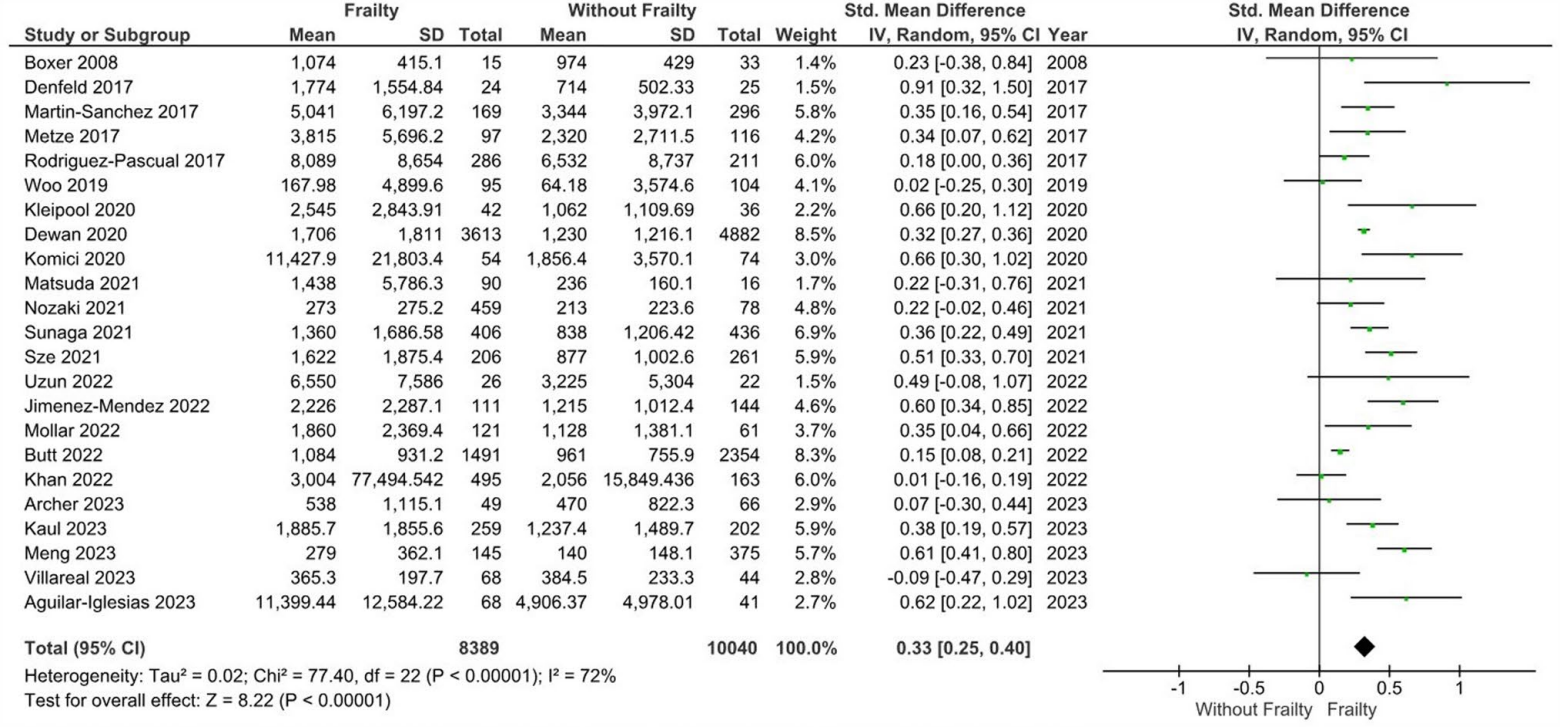
Mean differences in NT-proBNP levels according to frailty status in HF patients. Mean differences are presented with 95% confidence intervals using random effects model

Our main analysis showed that patients with HF and frailty (*n* = 8389) had significantly higher levels of NT-proBNP vs. those without frailty (*n* = 10,040) with a moderate degree of heterogeneity (*k* = 23; SMD: 0.33, 95%CI 0.25–0.40, I^2^ = 72%, *P* < 0.01) (Fig. 3). Subgroup analysis based on Fried (*k* = 11; SMD: 0.38, 95%CI 0.26–0.50, I^2^ = 44%, *P* < 0.01), FRAIL scale (*k* = 5; SMD: 0.28, 95%CI 0.01–0.54, I^2^ = 75%, *P* = 0.04), CFS criteria (*k* = 3; SMD: 0.44, 95%CI 0.30–0.57, I^2^ = 24%, *P* < 0.01), and the Rockwood index (*k* = 3; SMD: 0.18, 95%CI 0.02–0.33, I^2^ = 92%, *P* = 0.03) (Figure S5) demonstrated similar results. Our sensitivity analysis excluding studies in which patients with frailty had increased reported comorbidities revealed identical findings (*k* = 7; SMD: 0.39, 95%CI 0.22–0.56, I^2^ = 41%, *P* < 0.01) (Figure S6), while when we evaluated similar health status alongside similar frailty definition criteria (Fried criteria in this case), statistically significant differences were also observed (*k* = 6; SMD: 0.32, 95%CI 0.16–0.48, I^2^ = 23%, *P* < 0.01) (Figure S7). Sensitivity analysis based on studies with a high risk of bias did not alter the findings of the main analysis (*k* = 15; SMD: 0.30, 95%CI 0.20–0.40, I^2^ = 68%, *P* < 0.01) (Figure S8).

### CRP levels in patients with heart failure and frailty versus without frailty

**Fig. 4:**
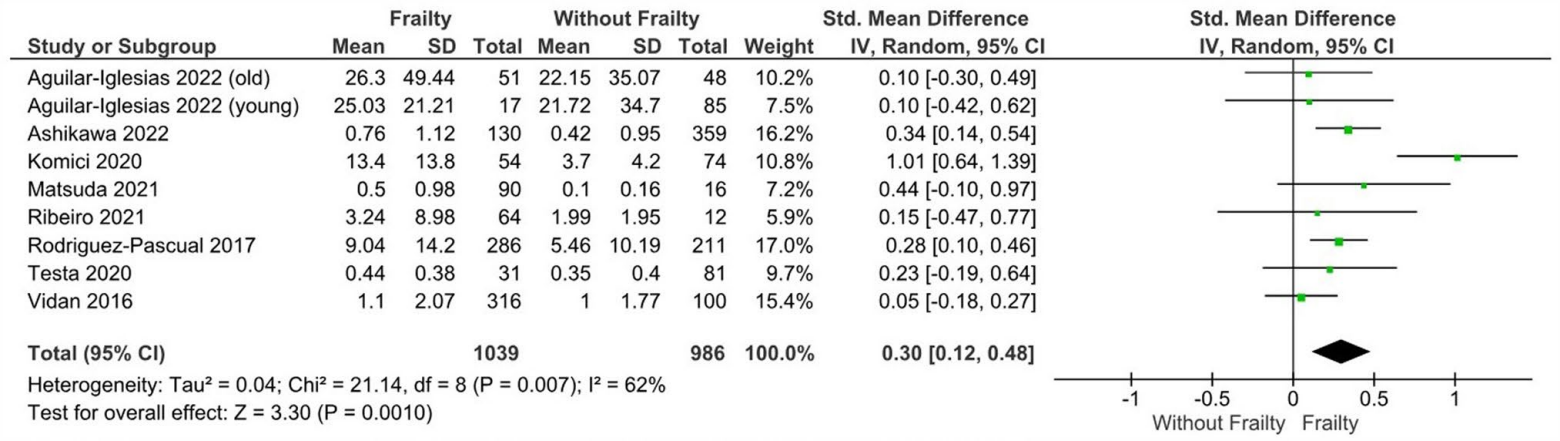
Mean differences in CRP levels according to frailty status in HF patients. Mean differences are presented with 95% confidence intervals using random effects model

Patients with HF and frailty (n = 1039) had significantly higher levels of CRP vs. those without frailty (n = 986) with a moderate degree of heterogeneity (*k* = 8; SMD: 0.30, 95%CI 0.12–0.48, I^2^ = 62%, *P* < 0.01) (Fig. 4).

For this analysis, the younger and older patients with HF in the study conducted by Aguilar-Iglesias et al*.* (2022) [32] were both included in the analysis and there was no overlap of participants between groups. In addition, subgroup analysis based on Fried (*k* = 5; SMD: 0.24, 95%CI 0.13–0.35, I^2^ = 0%, *P* < 0.01) and CFS criteria (*k* = 2; SMD: 0.76, 95%CI 0.20–1.32, I^2^ = 67%, *P* < 0.01) depicted identical results, but insignificant differences between groups were found when the FRAIL scale was used solely based on the younger and older patients of the Aguilar-Iglesias et al*.* (2022) study [32] (SMD: 0.10, 95%CI  – 0.22 to 0.41, I^2^ = 0%, *P* = 0.54) (Figure S9). Our sensitivity analysis excluding studies in which patients between groups had an increased number of comorbidities did not alter the findings of our main analysis (k = 6; SMD: 0.32, 95%CI 0.09–0.55, I2 = 75%, *P* < 0.01) (Figure S10). Sensitivity analysis based on studies with a high risk of bias did not alter the findings from the main analysis (*k* = 5; SMD: 0.25, 95%CI 0.13–0.36, I^2^ = 9%, *P* < 0.01) (Figure S11).

### NYHA levels in patients with heart failure and frailty versus without frailty

**Fig. 5:**
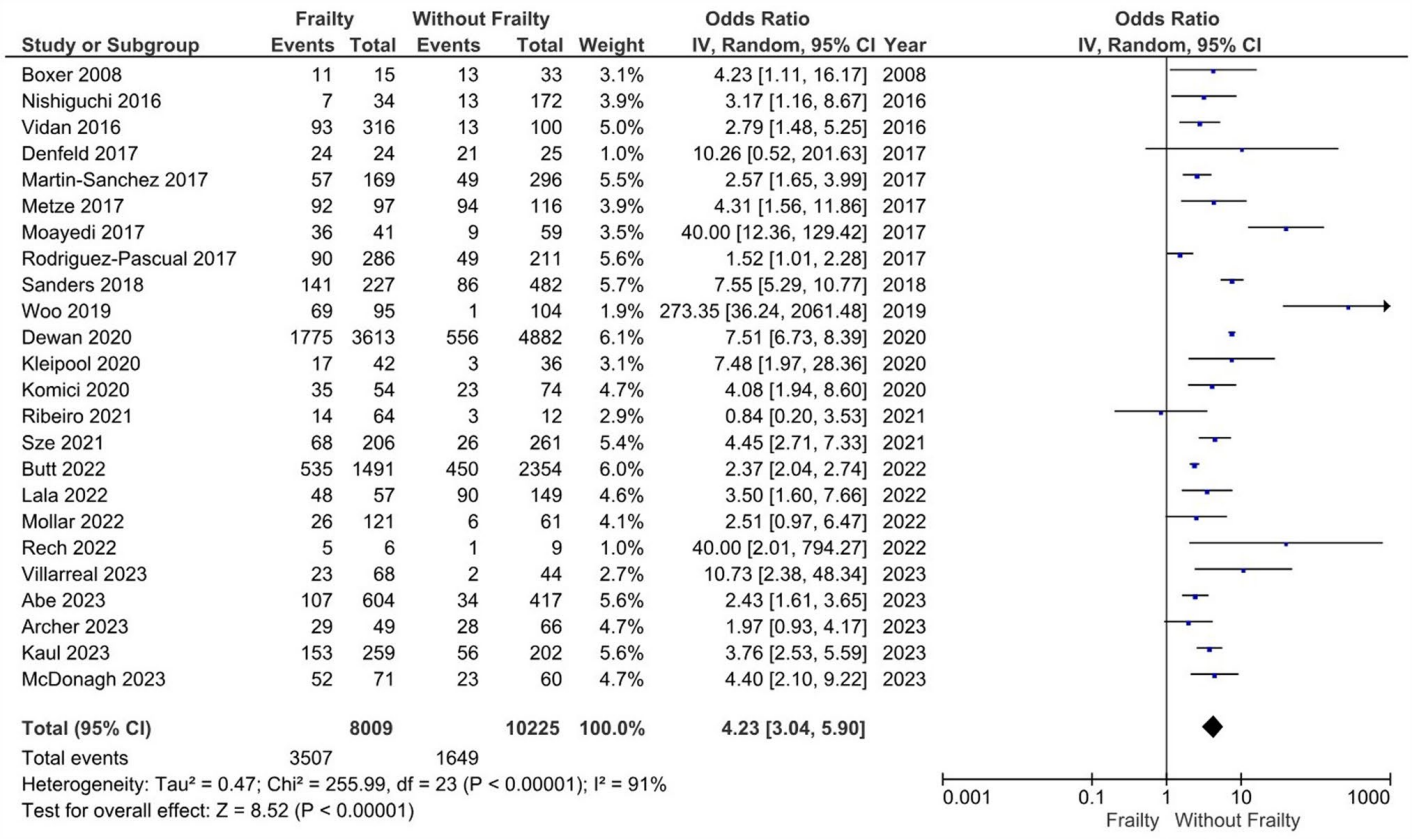
Odds ratios of NYHA classification score according to frailty status in HF patients. Odds ratios are presented with 95% confidence intervals using random effects model

Patients with HF and frailty (*n* = 8009) have a significantly increased risk of higher NYHA classification score vs. patients without frailty (*n* = 10,225) with a high degree of heterogeneity (*k* = 24; OR: 4.23, 95%CI 3.04–5.90, I^2^ = 91%, *P* < 0.01) (Fig. 5). Subgroup analysis based on Fried (*k* = 17; OR: 3.28, 95%CI 2.40–4.49, I^2^ = 63%, *P* < 0.01), FRAIL scale (*k* = 2; OR: 50.35, 95%CI 2.12–1197.89, I^2^ = 84%, *P* = 0.02), CFS criteria (*k* = 2; OR: 4.34, 95%CI 2.87–6.56, I^2^ = 0%, *P* < 0.01), and Rockwood index (*k* = 3; OR: 5.09, 95%CI 2.15–12.08, I^2^ = 99%, *P* < 0.01) similarly showed statistically significant results (Figure S12). Sensitivity analysis based on studies with high risk of bias did not alter the findings from the main analysis (*k* = 14; OR: 3.40, 95%CI 2.58–4.47, I^2^ = 72%, *P* < 0.01) (Figure S13).

### Meta-regression analyses

Variance among studies for the differences observed in BNP levels was detected in relation to age and BMI (*P* < 0.01), while in NT-proBNP levels, differences were observed only due to age (*P* < 0.01). In addition, age (*P* = 0.04) and BMI (*P* = 0.047) mediated the response of the association with CRP between groups, whereas for NYHA classification changes, age (*P* < 0.01) and LVEF% (*P* = 0.01) were significant moderators (Table S1).

### Risk of bias

Of the included studies, 13 studies were considered of having a high risk of bias [7, 31, 32, 39, 41, 43, 46, 52, 53, 60, 61, 63, 64], 16 as moderate risk of bias [18, 29, 33, 36, 37, 42, 44, 45, 48–50, 55, 56, 58, 59, 62], and 12 studies had a low risk [28, 30, 34, 35, 38, 40, 47, 51, 54, 57, 65, 66] (Table S2).

## Discussion

In this present study, we found that frailty is associated with higher levels of BNP, NT-proBNP, and CRP concentrations, and a worsened functional class (NYHA III/IV) in patients with HF. Age and BMI were covariates that mediated this relationship, partially explaining the aforementioned findings.

### Natriuretic peptides and frailty

The prevalence of frailty in individuals with HF is notably elevated, as a result of a common association between frailty and HF, sharing several risk factors [67]. The concurrent progression of these conditions involves shared mechanisms, including systemic inflammation, a higher burden of comorbidities, and abnormal skeletal muscle function and structure [68]. Sarcopenia is highly prevalent in patients with chronic HF, who are predisposed to skeletal muscle atrophy, accompanied by a relatively high proportion of non-muscular constituents such as intramuscular adipose tissue or fibrosis, exacerbating frailty [68]. In addition to the recognized utility of both BNP and NT-proBNP as diagnostic and prognostic indicators for HF patients, these biomarkers have been elucidated to be associated with frailty severity [7, 69, 70].

Our study demonstrates significantly elevated levels of BNP/NT-proBNP in patients with HF, as substantiated by several meta-analyses. In contrast to other meta-analyses focusing on the prevalence and prognostic impact of frailty in HF patients, our analysis extends to the association of BNP/NT-proBNP levels with frailty severity [71–73]. Li et al*.* (2023) highlighted BNP as an influential factor of frailty in older patients with HF, for which our data aligns with their findings. Interestingly, we identified age and BMI as potential covariates that may act as intermediates between frailty and elevated BNP, but not NT-proBNP. This may be explained, in part, due to a more pronounced link between altered BNP levels and adiposity, considering that NT-proBNP is not primarily degraded by natriuretic peptide receptors in adipose tissues [74, 75]. Furthermore, age was a common determinant in both BNP and NT-proBNP analyses, suggesting that older patients may be prone to increased BNP/NT-proBNP levels due to a higher burden of comorbidities such as renal function impairments [76, 77]. Although our subgroup analysis based on studies including commonly reported comorbidities between patients with and without frailty did not alter our observations, multiple studies did not assess for kidney or liver diseases that are contributors to elevated BNP and NT-proBNP concentrations to varied degrees [78, 79]. Lastly, elevation of BNP/NT-proBNP levels in frail patients was discernible across various frailty scales, despite the limitations of quick assessment scales used in clinical settings.

### CRP, NYHA class, and frailty

In the context of inflammation and frailty, chronic inflammation, characterized by higher oxidative stress and pro-inflammatory cytokines, is recognized as an important mechanism underpinning frailty, impacting multiple organs [67]. Neurohormonal factors activated in HF, such as the renin–angiotensin–aldosterone system, may further contribute to a pro-inflammatory state [80]. Elevation of CRP in patients with frailty has been well-documented, and our study further corroborated the significant elevation of CRP levels in a cohort of patients with HF and frailty. Our results also suggest that age and BMI could mediate this association, aligning with findings from previous meta-analyses [81, 82]. Moreover, symptoms of HF categorized by NYHA class, despite inherent subjectivity, remain fundamental. For instance, fatigue, a principal characteristic of frailty, complicates the differentiation of symptoms between frailty and HF. Meta-analyses have demonstrated that a preponderance of patients concomitantly exhibiting frailty and HF manifest elevated symptomatology (NYHA III/IV class), aligning with our findings of a significant association between frailty and NYHA class for each frailty score.

### Limitations

The inclusion of studies with a diverse age demographic may impact the extrapolation of results to studies predominantly comprised of older-aged cohorts, where elevated BNP/NT-proBNP levels may be influenced by comorbidities, which may had not been reported sufficiently in several trials. In addition, these results cannot be extrapolated in relation to a particular sex, considering that the prevalence of frailty is more pronounced in women compared to men [83]. Likewise, we did not differentiate between HF with reduced (HFrEF) and preserved (HFpEF) ejection fraction, that are characterized by different levels of natriuretic peptides, potentially displaying distinct outcomes linked to frailty. In addition, we were unable to ascertain the potential ramifications of hospitalized versus non-hospitalized patients, given the potential variations in settings, rehabilitation regimens, and severity of HF. Finally, our analyses relied on cross-sectional data, precluding the establishment of causal relationships.

## Conclusions

In conclusion, frailty in HF is linked to increased concentrations of BNP, NT-proBNP, and CRP, which have been epidemiologically associated with adverse outcomes. The increased risk of NYHA III/IV classification further emphasizes the clinical impact of frailty in this population.

### Supplementary Information

Below is the link to the electronic supplementary material.

Supplementary file1 (DOCX 2850 KB)

## Data Availability

Data are available upon request.
