# Supplementary material for: Association between natriuretic peptides and C-reactive protein with frailty in heart failure: a systematic review and meta-analysis
Source: Aging Clin Exp Res. 2024 Mar 6;36(1):57. doi: 10.1007/s40520-024-02713-x (PMC10917829; doi:10.1007/s40520-024-02713-x)
Supplement: Supplementary file 1 — Supplementary file1 (DOCX 2850 KB) [file 40520_2024_2713_MOESM1_ESM.docx]

Table of Contents

Meta-regression analyses1

Risk of bias assessment2

Subgroup and sensitivity analyses5

**Meta regression analyses**

**Table S1.** Meta-regression analyses of patients with heart failure and frailty vs. patients with heart failure without frailty.

| **Confounder** | *r* | SE | 95%CI | *z* | P |
| --- | --- | --- | --- | --- | --- |
| **BNP** |  | | | | |
| Age | 9.560 | 2.879 | 3.92 – 15.20 | 3.32 | <0.01* |
| BMI | -1.664 | 0.338 | -2.33 – -1.00 | -4.93 | <0.01* |
| LVEF% | -0.443 | 1.586 | -3.55 – 2.67 | -0.28 | 0.780 |
| **NT-proBNP** |  | | | | |
| Age | 6.419 | 1.238 | 3.99 – 8.84 | 5.19 | <0.01* |
| BMI | 0.426 | 0.542 | -0.64 – 1.49 | 0.79 | 0.432 |
| LVEF% | 1.071 | 0.847 | -0.59 – 2.73 | 1.26 | 0.206 |
| **CRP** |  |  |  |  |  |
| Age | 6.196 | 3.014 | 0.29 – 12.10 | 2.06 | 0.04* |
| BMI | -1.418 | 0.714 | -2.82 – -0.02 | -1.98 | 0.047* |
| LVEF% | -1.884 | 1.844 | -5.50 – 1.73 | -1.02 | 0.307 |
| **NYHA** |  |  |  |  |  |
| Age | 3.580 | 0.539 | 2.52 – 4.64 | 6.64 | <0.01* |
| BMI | 0.681 | 0.914 | -1.11 – 2.47 | 0.75 | 0.456 |
| LVEF% | 2.186 | 0.844 | 0.53 – 3.84 | 2.59 | 0.01* |

*Indicates significance.
BMI, body mass index; BNP, brain natriuretic peptide; CRP, c-reactive protein; LVEF, left ventricular ejection fraction; NT-proBNP, N-terminal-pro B-type natriuretic peptide; NYHA, New York Heart Association.

**Risk of bias assessment**

**Table S2.** Quality assessment of the included studies based on the Methodological Index for Non-Randomized Studies (MINORS) tool.

|  |  |  |  |  |  |  |  |  |  |  |
| --- | --- | --- | --- | --- | --- | --- | --- | --- | --- | --- |
| Author, Year | **Aim** | **Inclusion of consecutive patients** | **Prospective collection of data** | **Endpoints appropriate to the aim of the study** | **Unbiased assessment of the study endpoint** | **Follow-up period appropriate to the aim of the study** | **Loss to follow up less than 5%** | **Prospective calculation of the study size** | **Total** | **Risk of bias** |
| Abe, 2023 | 2 | 1 | 2 | 2 | 0 | 2 | 2 | 0 | 11 | Low |
| Aguilar-Iglesias, 2022 | 2 | 1 | 0 | 1 | 0 | 0 | 0 | 0 | 4 | High |
| AGUILAR-IGLESIAS, 2023 | 2 | 1 | 1 | 2 | 0 | 0 | 0 | 2 | 8 | Moderate |
| Archer, 2023 | 2 | 1 | 0 | 1 | 0 | 0 | 0 | 0 | 4 | High |
| Ashikawa, 2023 | 2 | 1 | 2 | 2 | 0 | 2 | 0 | 0 | 9 | Moderate |
| BOXER, 2008 | 2 | 1 | 0 | 0 | 0 | 0 | 0 | 0 | 3 | High |
| butt, 2022 | 2 | 1 | 2 | 2 | 2 | 2 | 0 | 0 | 11 | Low |
| DENFELD, 2017 | 2 | 0 | 1 | 1 | 0 | 0 | 1 | 0 | 5 | High |
| DEWAN, 2020 | 1 | 1 | 0 | 1 | 2 | 1 | 0 | 0 | 6 | High |
| jIMENEZ-MENDEZ, 2022 | 2 | 2 | 2 | 2 | 0 | 2 | 2 | 0 | 12 | Low |
| kANENAWA, 2021 | 2 | 0 | 1 | 1 | 0 | 2 | 2 | 0 | 8 | Moderate |
| kAUL, 2023 | 2 | 1 | 0 | 1 | 0 | 2 | 2 | 0 | 8 | Moderate |
| KHAN, 2022 | 2 | 0 | 0 | 2 | 2 | 2 | 2 | 2 | 12 | Low |
| KLEIPOOL, 2020 | 2 | 2 | 2 | 1 | 0 | 1 | 0 | 0 | 8 | Moderate |
| komici, 2020 | 1 | 2 | 0 | 1 | 0 | 0 | 0 | 0 | 6 | High |
| Kondo, 2023 | 2 | 1 | 2 | 1 | 0 | 2 | 0 | 0 | 8 | Moderate |
| KUSUNOSE, 2018 | 2 | 2 | 2 | 2 | 2 | 2 | 2 | 0 | 14 | Low |
| lala, 2022 | 2 | 1 | 2 | 1 | 0 | 2 | 2 | 0 | 10 | Low |
| Martin-sanchez, 2017 | 2 | 1 | 0 | 1 | 0 | 2 | 2 | 0 | 8 | Moderate |
| MATSUDA, 2021 | 2 | 2 | 0 | 1 | 0 | 2 | 2 | 0 | 9 | Moderate |
| McDonagh, 2023 | 2 | 2 | 1 | 1 | 0 | 2 | 2 | 0 | 10 | Low |
| MENG, 2023 | 2 | 1 | 1 | 1 | 0 | 2 | 0 | 0 | 7 | High |
| METZE, 2017 | 2 | 1 | 0 | 1 | 0 | 2 | 2 | 0 | 8 | Moderate |
| MOAYEDI, 2017 | 2 | 1 | 0 | 1 | 0 | 2 | 2 | 0 | 8 | Moderate |
| mollar, 2022 | 2 | 2 | 0 | 1 | 0 | 2 | 2 | 0 | 9 | Moderate |
| nishiguchi, 2016 | 2 | 2 | 0 | 1 | 0 | 0 | 0 | 0 | 5 | High |
| NOZAKI, 2020 | 2 | 2 | 0 | 2 | 0 | 0 | 0 | 0 | 6 | High |
| NOZAKI, 2021 | 2 | 1 | 0 | 1 | 0 | 2 | 2 | 0 | 8 | Moderate |
| PANDEY, 2022 | 2 | 2 | 2 | 2 | 0 | 2 | 0 | 0 | 10 | Low |
| RIBEIRO, 2021 | 2 | 2 | 0 | 1 | 0 | 0 | 0 | 2 | 7 | High |
| RODRIGUEZ-PASCUAL, 2017 | 2 | 1 | 0 | 1 | 0 | 2 | 2 | 0 | 8 | Moderate |
| RECH, 2022 | 2 | 0 | 0 | 2 | 0 | 2 | 0 | 0 | 6 | High |
| SANDERS, 2018 | 2 | 0 | 1 | 1 | 0 | 0 | 0 | 0 | 4 | High |
| SUNAGA, 2021 | 2 | 0 | 2 | 2 | 0 | 1 | 2 | 0 | 9 | Moderate |
| SZE, 2021 | 2 | 2 | 1 | 2 | 1 | 1 | 1 | 0 | 11 | Low |
| TESTA, 2020 | 2 | 2 | 2 | 2 | 0 | 2 | 0 | 0 | 10 | Low |
| UZUN, 2022 | 2 | 2 | 2 | 1 | 0 | 1 | 0 | 0 | 8 | Moderate |
| VIDAN, 2016 | 2 | 2 | 2 | 2 | 1 | 1 | 1 | 0 | 11 | Low |
| VILLAREAL, 2023 | 2 | 0 | 2 | 1 | 0 | 2 | 0 | 2 | 9 | Moderate |
| WANG, 2023 | 2 | 2 | 2 | 2 | 0 | 2 | 0 | 0 | 10 | Low |
| WOO, 2019 | 2 | 0 | 1 | 2 | 0 | 1 | 0 | 0 | 6 | High |
|  |  |  |  |  |  |  |  |  |  |  |

**Subgroup and sensitivity analyses**


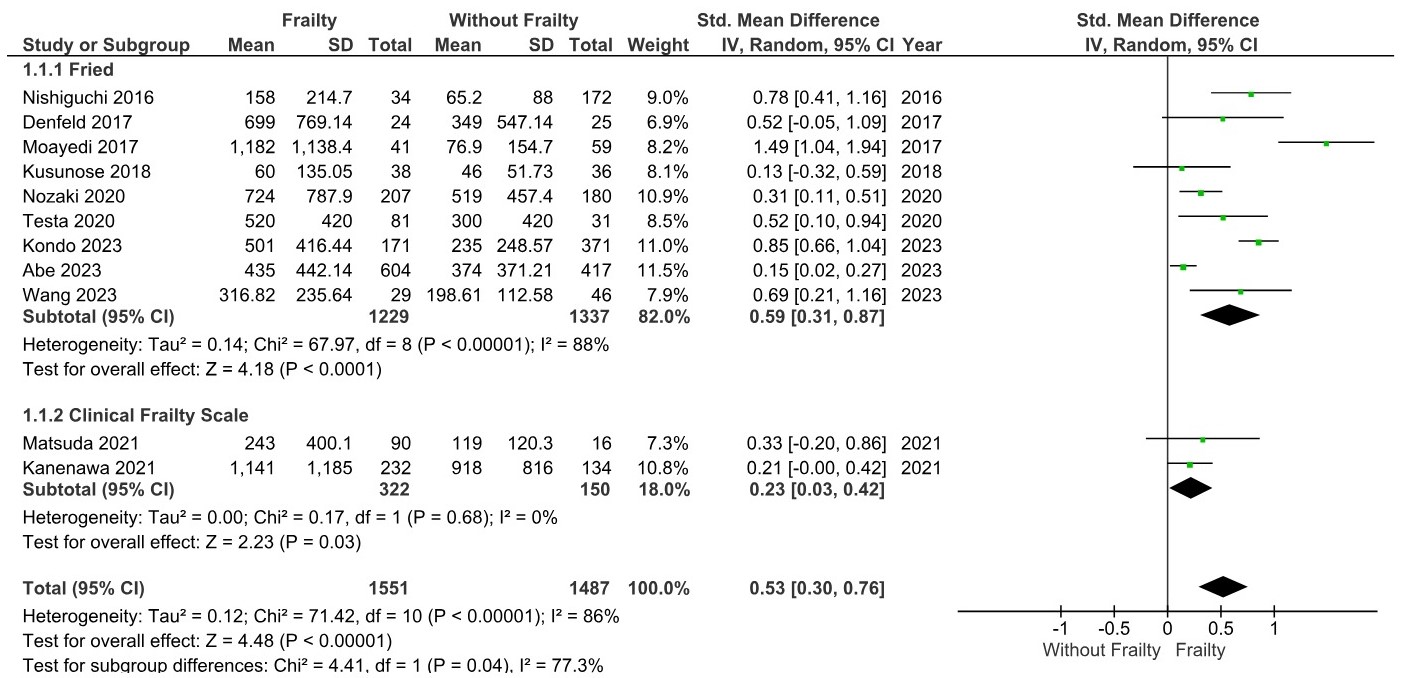
**Figure S1**. Subgroup analysis of BNP variations by frailty in heart failure patients using Fried and Clinical Frailty Scale criteria. Mean differences are presented with 95% confidence intervals using random effects model.


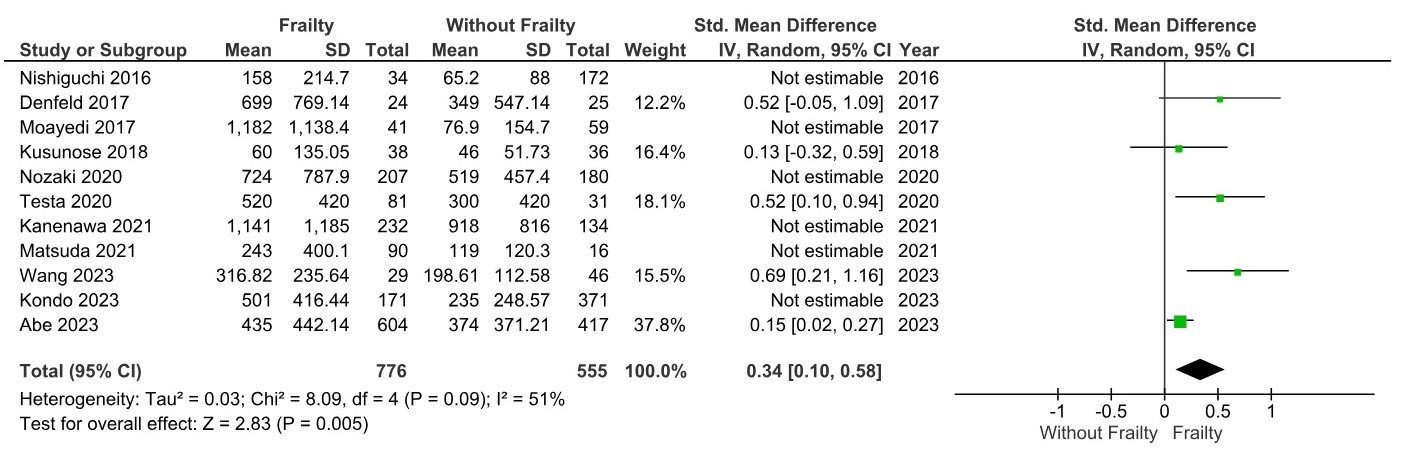
**Figure S2.** Mean differences in BNP levels according to frailty status in heart failure patients after exclusion of studies with increased prevalence of reported comorbidities in frail patients. Mean differences are presented with 95% confidence intervals using random effects model.


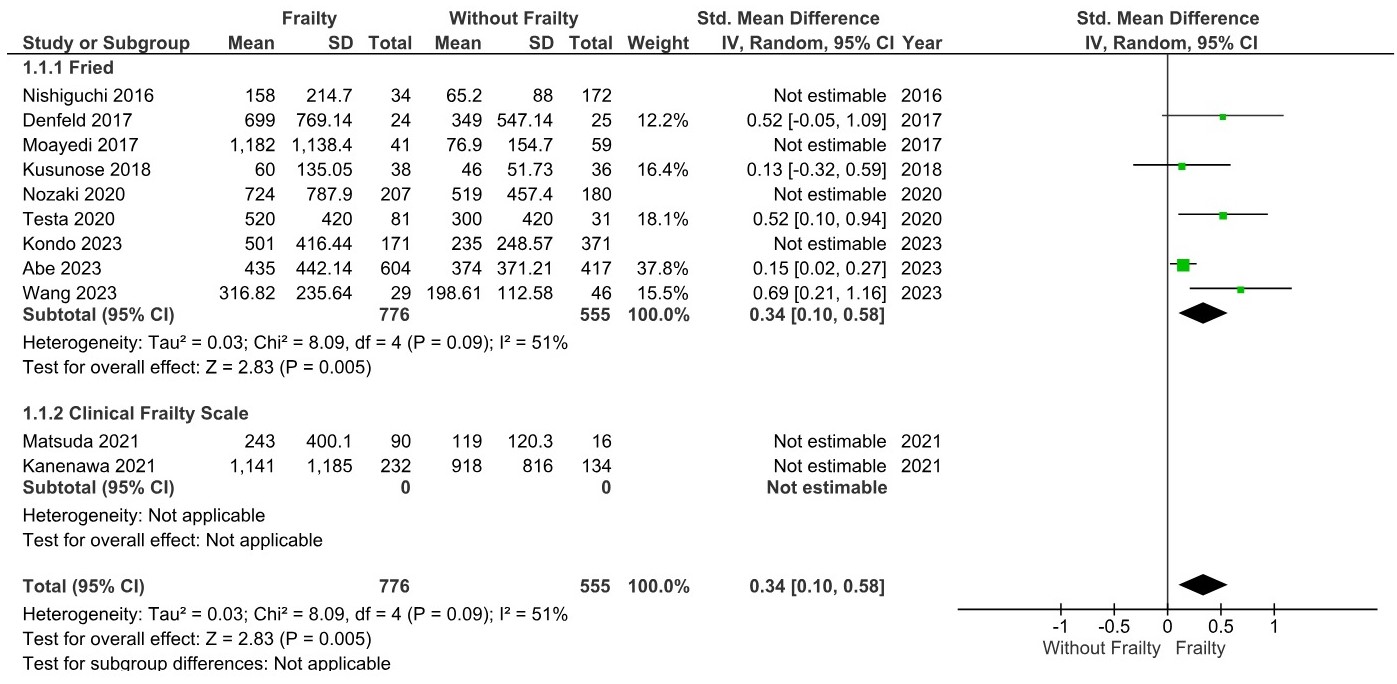
**Figure S3.** Subgroup analysis of BNP variations by frailty in heart failure patients using Fried and Clinical Frailty Scale criteria after exclusion of studies with increased prevalence of reported comorbidities in frail patients. Mean differences are presented with 95% confidence intervals using random effects model.

**
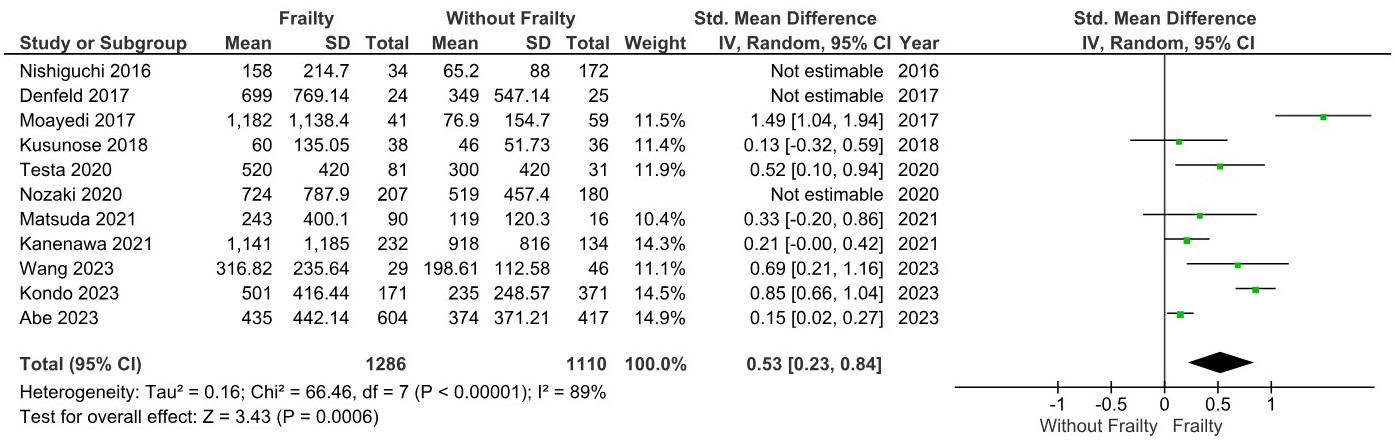
**

**Figure S4.** Mean differences in BNP levels according to frailty status in heart failure patients after exclusion of studies with high risk of bias. Mean differences are presented with 95% confidence intervals using random effects model.

**
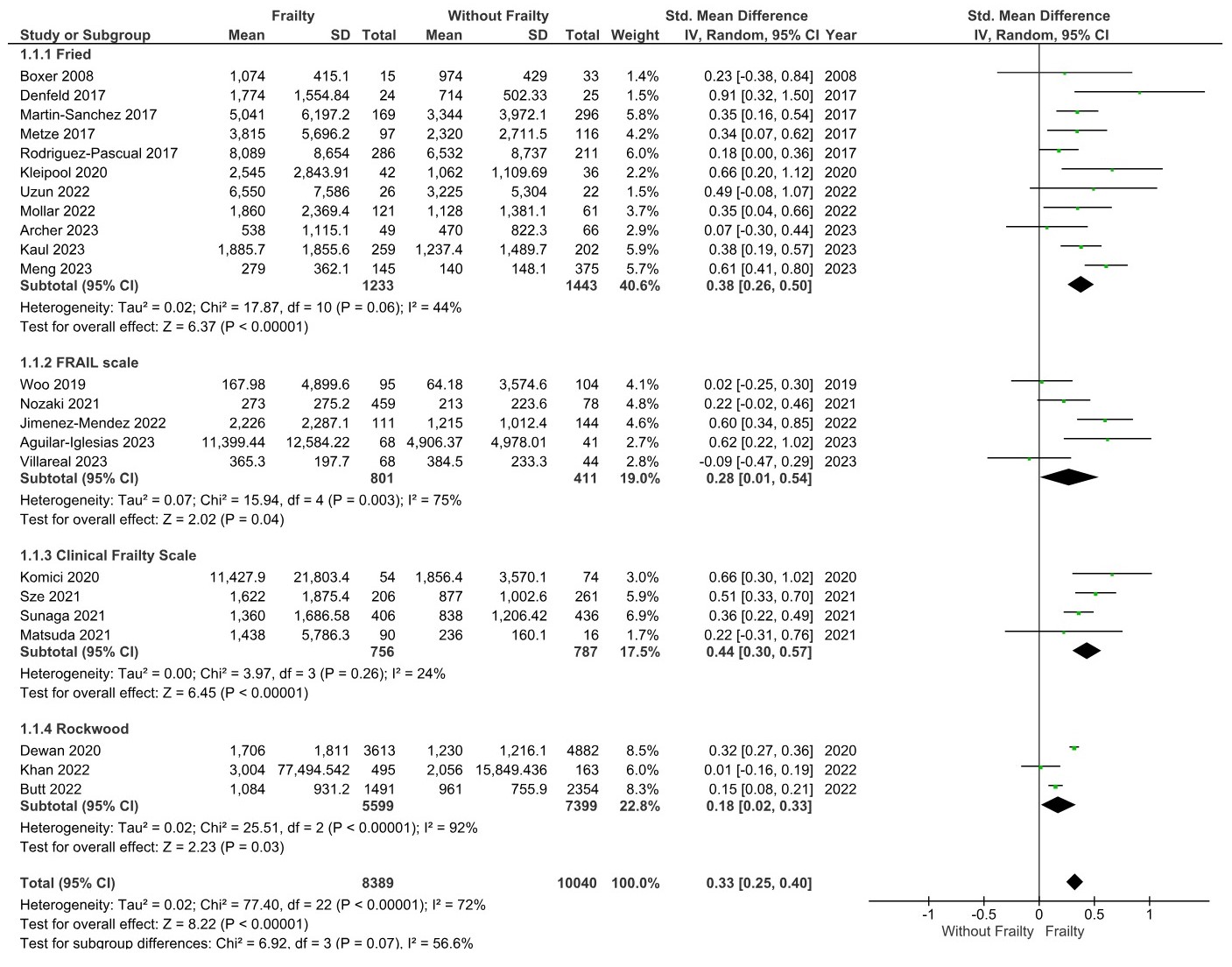
**

**Figure S5.** Subgroup analysis of NT-proBNP variations by frailty in heart failure patients using Fried, FRAIL scale, Clinical Frailty Scale criteria and the Rockwood index. Mean differences are presented with 95% confidence intervals using random effects model.

**
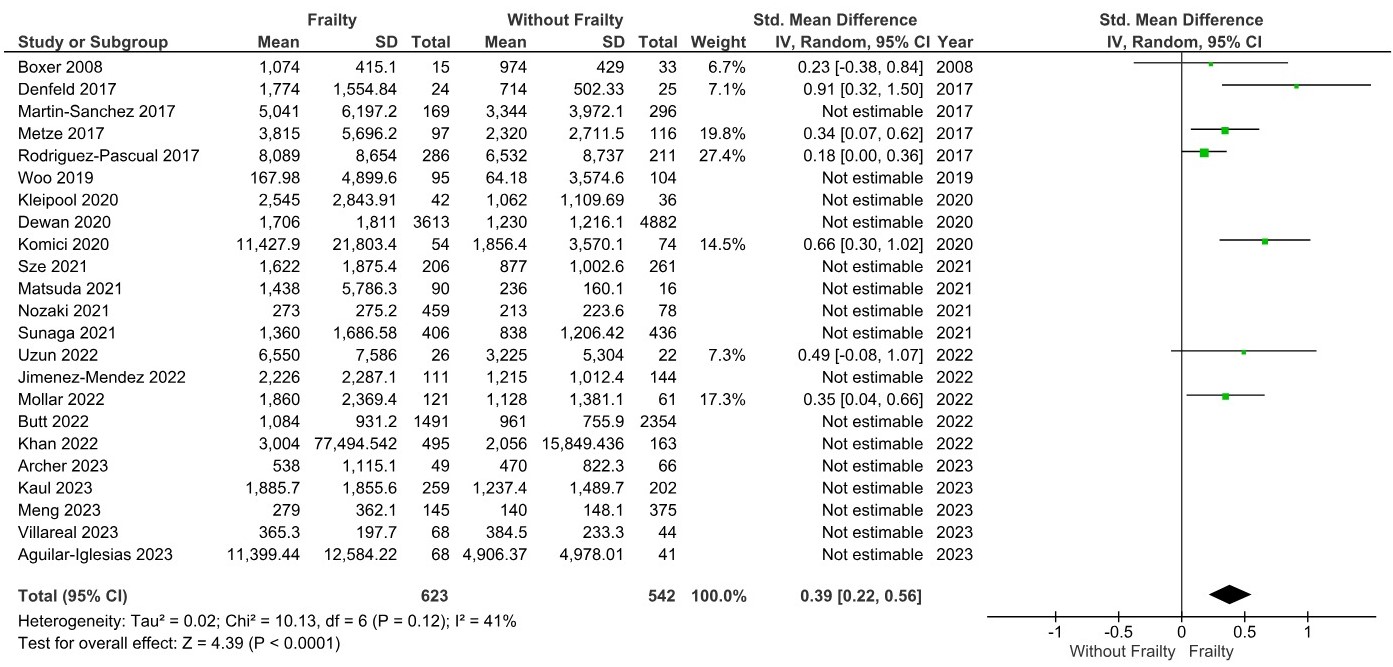
**

**Figure S6.** Mean differences in NT-proBNP levels according to frailty status in heart failure patients after exclusion of studies with increased prevalence of reported comorbidities in frail patients. Mean differences are presented with 95% confidence intervals using random effects model.

**
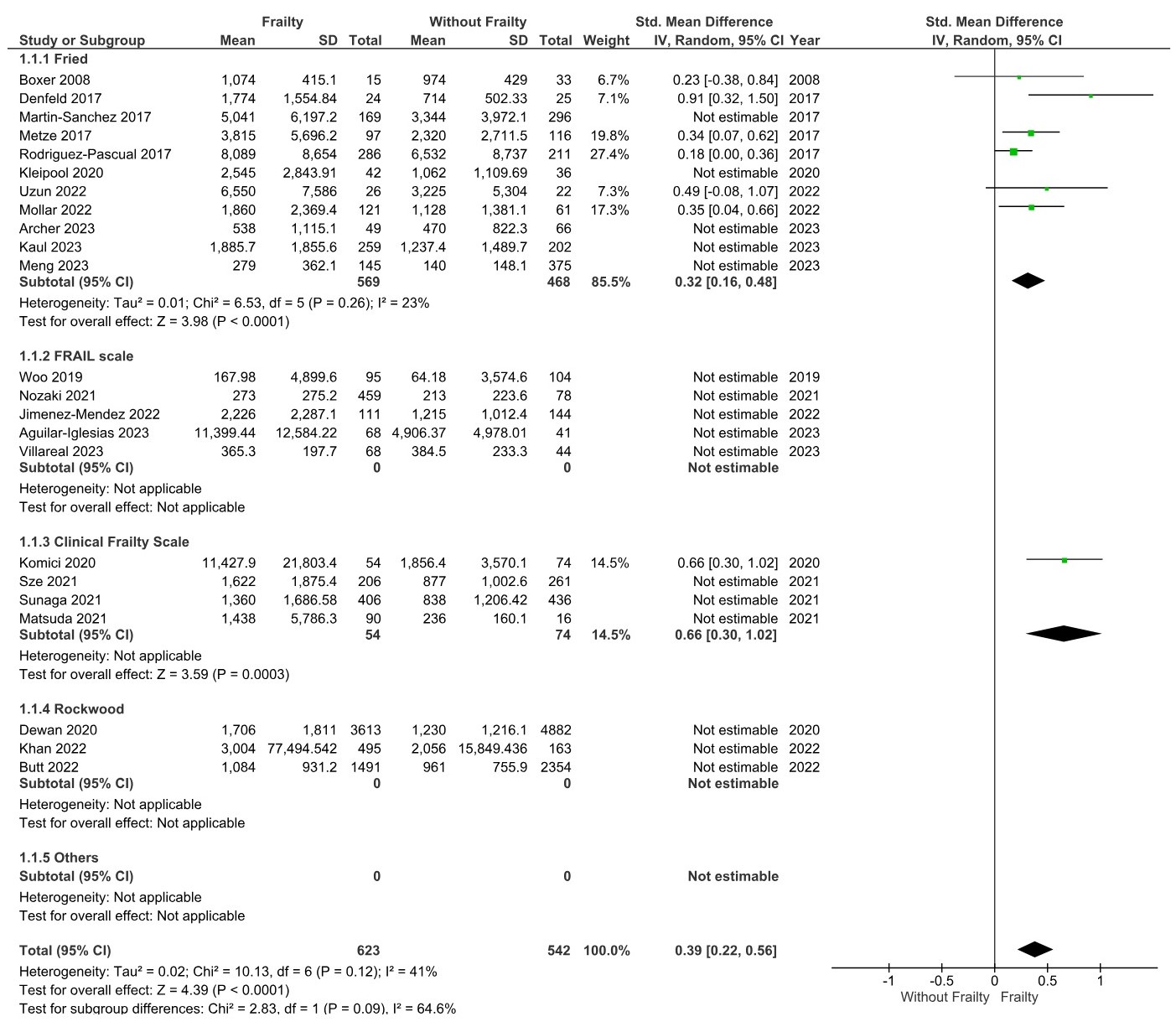
**

**Figure S7.** Subgroup analysis of NT-proBNP variations by frailty in heart failure patients using Fried, FRAIL scale, Clinical Frailty Scale criteria and the Rockwood index after exclusion of studies with increased prevalence of reported comorbidities in frail patients. Mean differences are presented with 95% confidence intervals using random effects model.

**
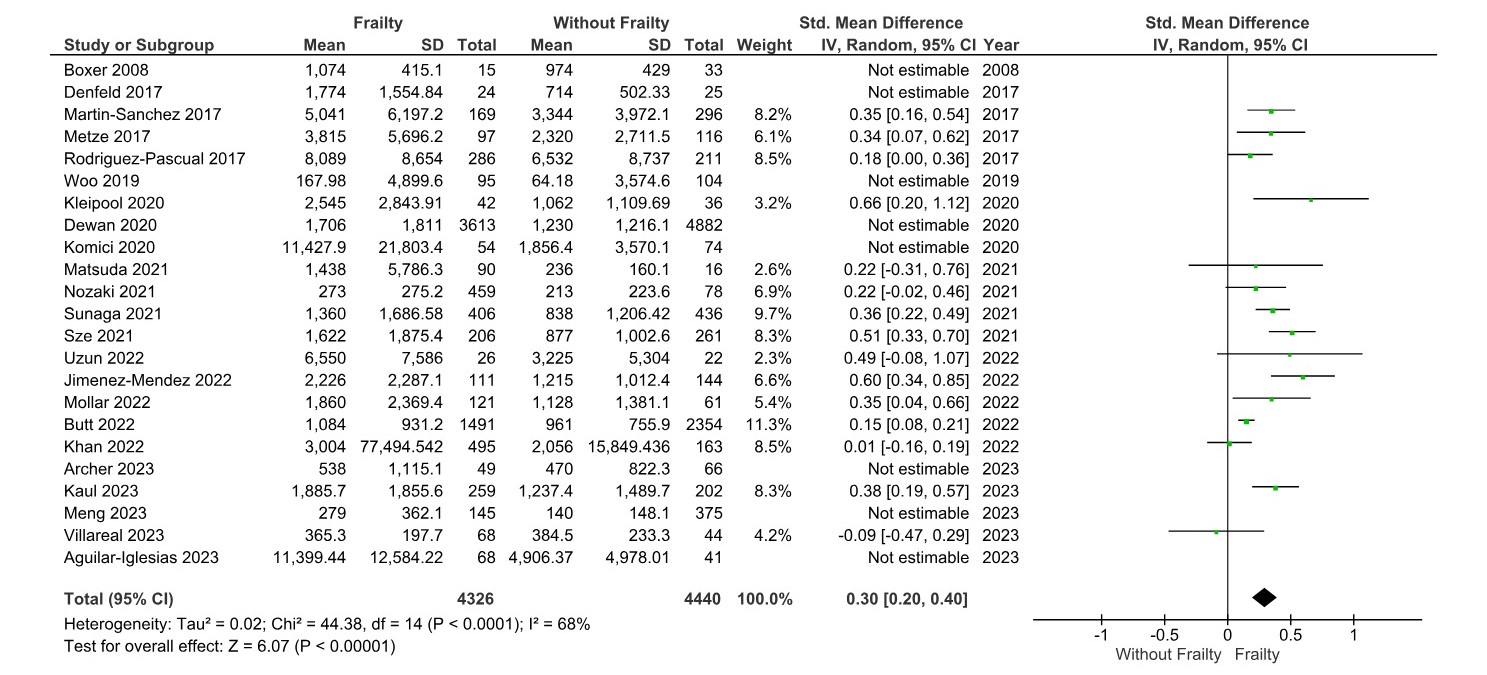
**

**Figure S8.** Mean differences in NT-proBNP levels according to frailty status in heart failure patients after exclusion of studies with high risk of bias. Mean differences are presented with 95% confidence intervals using random effects model.

**
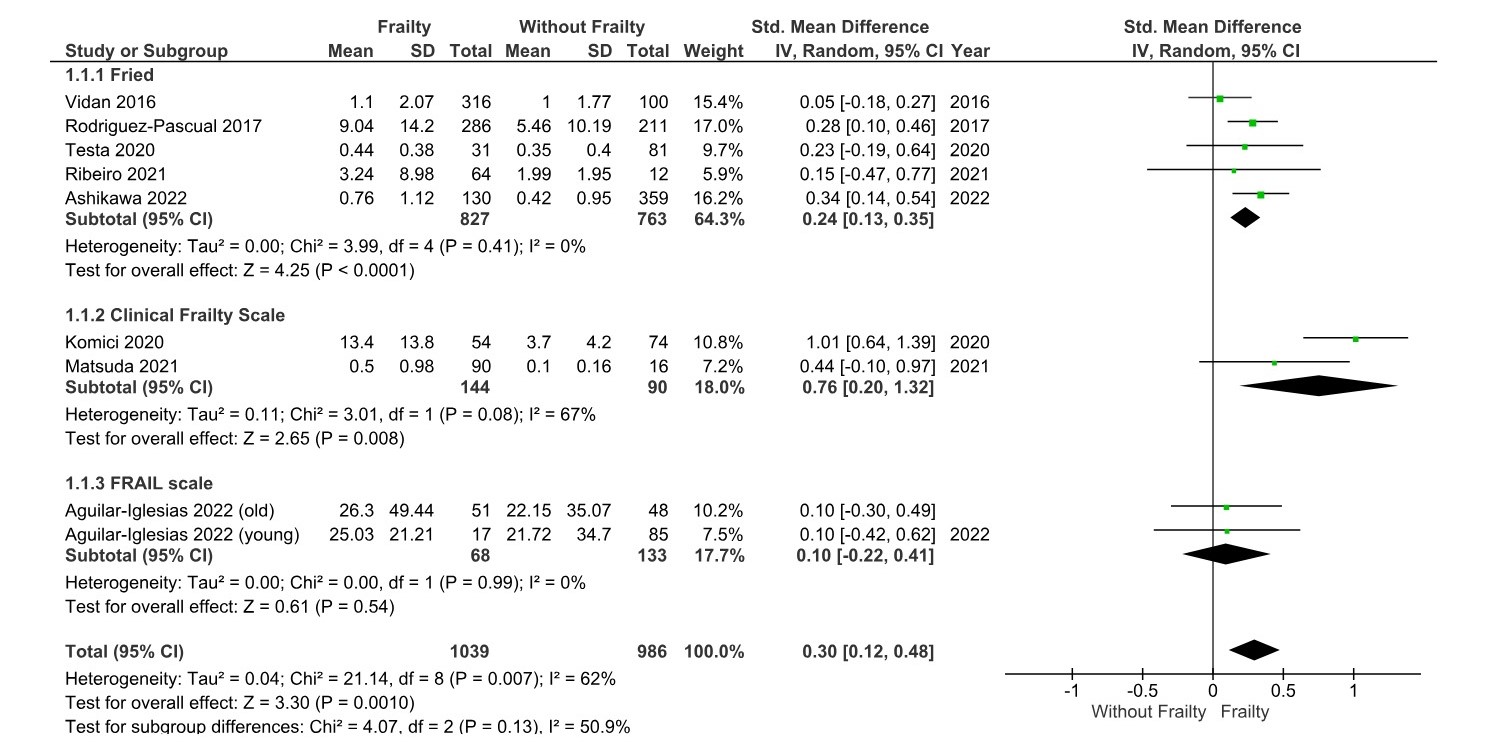
**

**Figure S9.** Subgroup analysis of CRP variations by frailty in heart failure patients using Fried, FRAIL scale, Clinical Frailty Scale criteria and the Rockwood index. Mean differences are presented with 95% confidence intervals using random effects model.

**
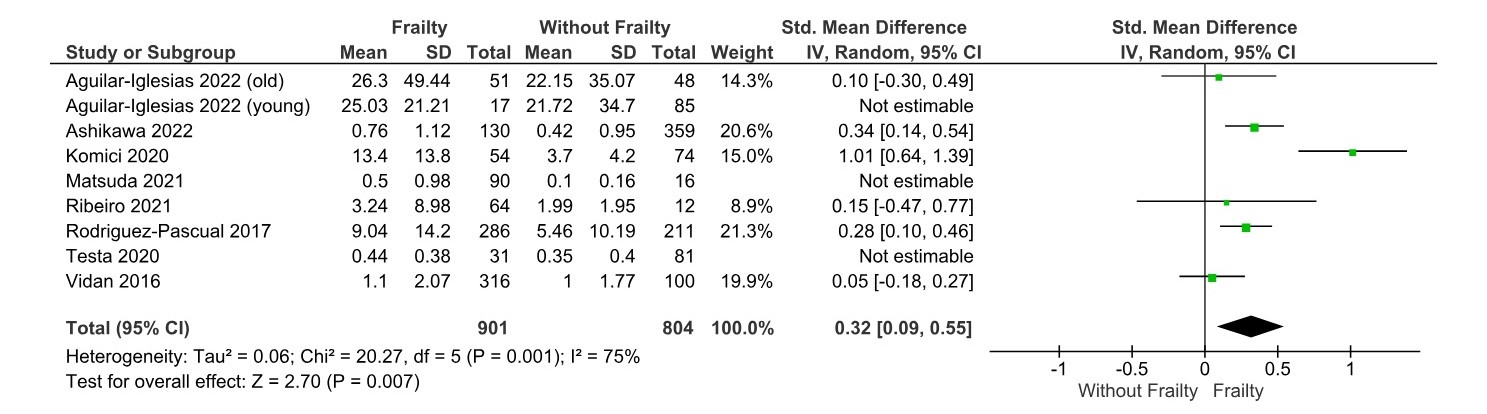
**

**Figure S10.** Mean differences in CRP levels according to frailty status in heart failure patients after exclusion of studies with increased prevalence of reported comorbidities in frail patients. Mean differences are presented with 95% confidence intervals using random effects model.

**
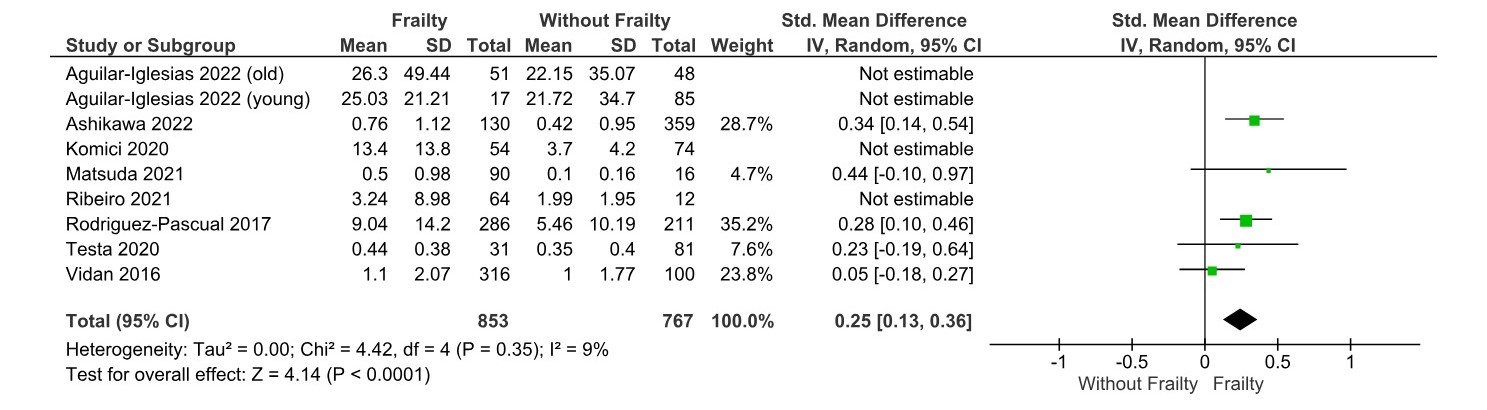
**

**Figure S11.** Mean differences in CRP levels according to frailty status in heart failure patients after exclusion of studies with high risk of bias. Mean differences are presented with 95% confidence intervals using random effects model.

**
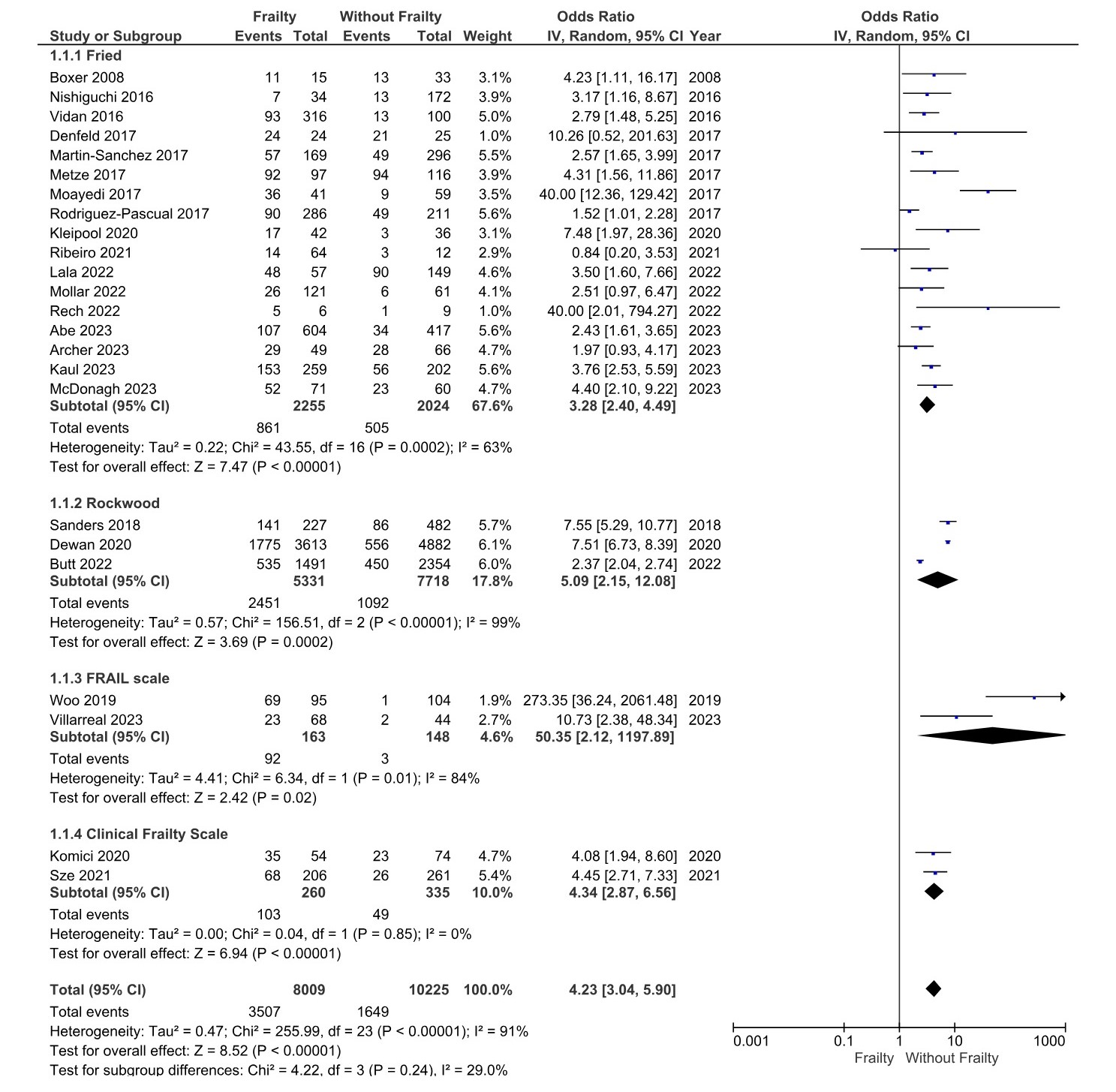
**

**Figure S12.** Subgroup analysis of NYHA levels variations by frailty in heart failure patients using Fried, FRAIL scale, Clinical Frailty Scale criteria and the Rockwood index. Odds ratios are presented with 95% confidence intervals using random effects model.

**
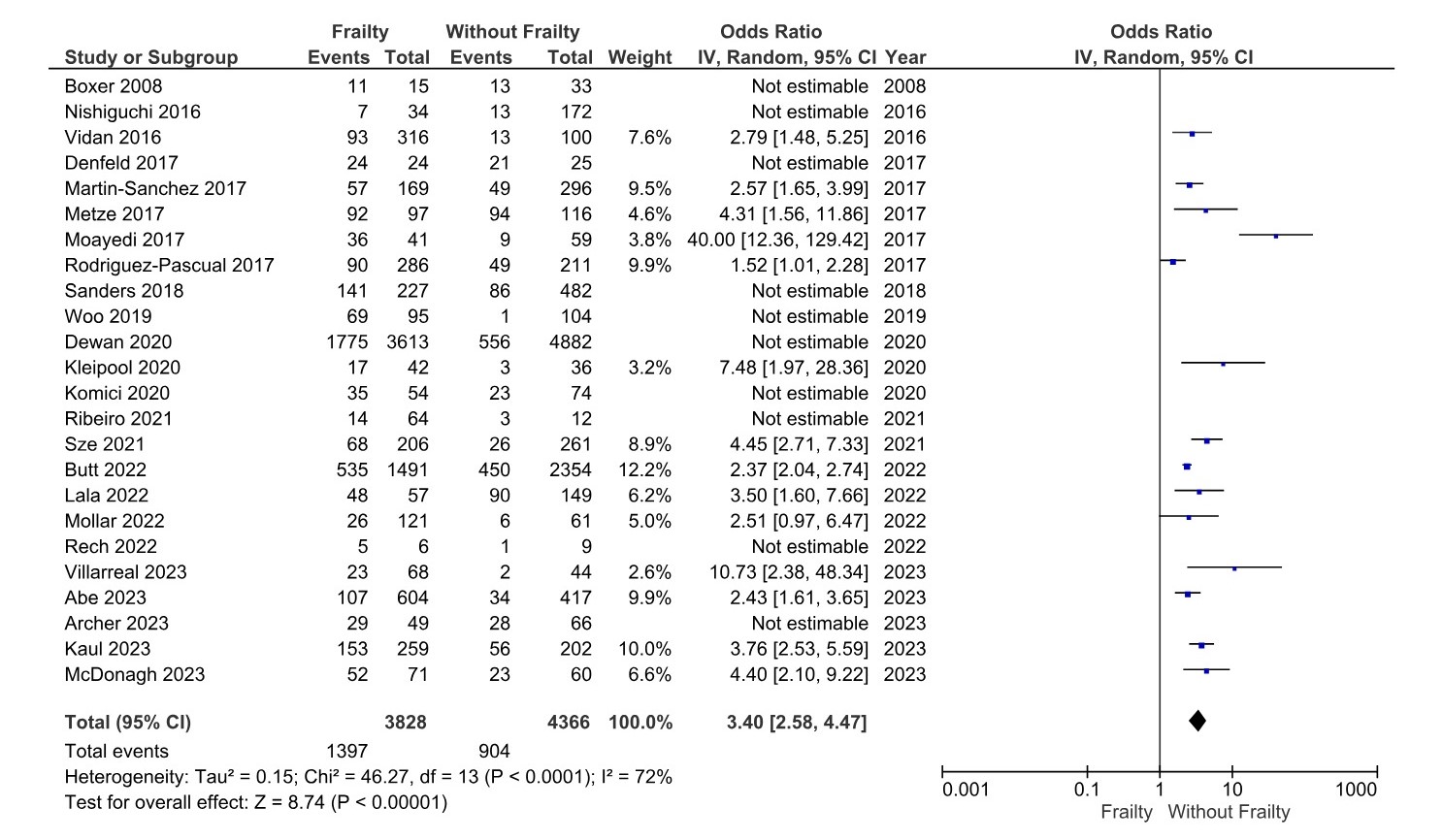
**

**Figure S13.** Odds ratios of NYHA classification score according to frailty status in heart failure patients after exclusion of studies with high risk of bias. Odds ratios are presented with 95% confidence intervals using random effects model.
